# Tiny Machine Learning and On-Device Inference: A Survey of Applications, Challenges, and Future Directions

**DOI:** 10.3390/s25103191

**Published:** 2025-05-19

**Authors:** Soroush Heydari, Qusay H. Mahmoud

**Affiliations:** Department of Electrical, Computer and Software Engineering, Ontario Tech University, Oshawa, ON L1G 0C5, Canada

**Keywords:** TinyML, IoT, sensors, edge AI, edge computing, embedded ML, embedded systems, resource-constrained devices

## Abstract

The growth in artificial intelligence and its applications has led to increased data processing and inference requirements. Traditional cloud-based inference solutions are often used but may prove inadequate for applications requiring near-instantaneous response times. This review examines Tiny Machine Learning, also known as TinyML, as an alternative to cloud-based inference. The review focuses on applications where transmission delays make traditional Internet of Things (IoT) approaches impractical, thus necessitating a solution that uses TinyML and on-device inference. This study, which follows the PRISMA guidelines, covers TinyML’s use cases for real-world applications by analyzing experimental studies and synthesizing current research on the characteristics of TinyML experiments, such as machine learning techniques and the hardware used for experiments. This review identifies existing gaps in research as well as the means to address these gaps. The review findings suggest that TinyML has a strong record of real-world usability and offers advantages over cloud-based inference, particularly in environments with bandwidth constraints and use cases that require rapid response times. This review discusses the implications of TinyML’s experimental performance for future research on TinyML applications.

## 1. Introduction

The rapid advancement of artificial intelligence (AI) applications has led to an increase in data generation and processing requirements. Traditional cloud-based approaches to AI processing offer considerable computational resources but show increasing limitations in meeting the demand for time-sensitive applications. Despite the enormous amounts of computing power available in cloud-hosted environments, executing all computing tasks in the cloud requires significant bandwidth and introduces considerable latency, which can impede success in real-time applications [1]. This is particularly true for time-sensitive applications where immediate response times are crucial such as in healthcare monitoring systems and autonomous vehicles. The Internet of Things (IoT) architecture’s reliance on cloud computing creates a computational bottleneck regarding latency and bandwidth needs that limits the potential of AI applications for scenarios where network connectivity may be unreliable, or bandwidth constraints exist.

**Figure 1:**
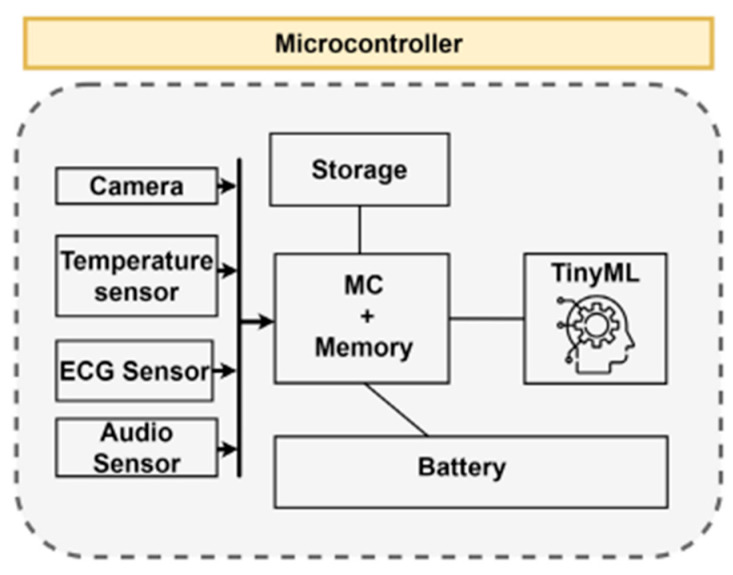
TinyML hardware schema from [1].

TinyML provides an alternative solution to cloud-based AI processing by changing the deployment and execution of machine learning models. TinyML enables ML tasks to be run on hardware with size and processing constraints, fundamentally changing the approach to edge computing [1]. The tradeoffs between cloud-hosted and on-device inference are particularly significant in time-critical applications. In autonomous vehicle systems, waiting for cloud-based decision making can have potentially catastrophic consequences, making local processing through TinyML a more viable option. TinyML offers near-zero latency for ML services by reducing the dependence on external communication, which is a crucial advantage in safety-critical systems. TinyML can also address concerns about data privacy and security, as inference is carried out from within the device rather than from cloud servers. The implementation of TinyML solutions requires careful consideration of hardware constraints, as typical microcontrollers have limited clock rates and memory sizes, requiring optimization to fit the model on the device while maintaining adequate model accuracy. Thus, the decision between cloud-hosted and on-device inference becomes a critical architectural choice that must consider latency requirements, privacy concerns, and computational capabilities against the specific demands of each application [2]. Figure 1 shows an example of how TinyML fits into a hardware device.

TinyML models are chosen based on the required task and the available hardware. The device must have sufficient memory to run the model and enough storage to store the trained model weights as well as the input data collected by the sensors.

TinyML’s use cases have become more valuable in low-resource settings where access to traditional IoT networks is limited, potentially requiring on-device processing capability. This paradigm shift has moved beyond the conventional approach of serving ML to IoT devices from the cloud, instead focusing on processing ML within the IoT device itself [3]. This reimagining of machine learning architecture involves considering the questions of model optimization, power consumption, and accuracy tradeoffs across different application domains. Surveys on TinyML emphasize how different use cases demand different balances between latency, power consumption, and model accuracy, particularly for mission-critical applications where the benefits of on-device processing must be considered against the limitations of embedded hardware [1].

Researchers may want to know whether TinyML inference can retain the same accuracy and efficiency as IoT inference in a controlled experiment. Practitioners may want to know whether TinyML is more applicable than IoT inference to image recognition in a sensor. For which applications, or at what scale, does IoT prove superior or does TinyML become unviable?

This study provides a state-of-the-art literature review of TinyML characteristics and applications for the purpose of studying on-device inference in TinyML for applications where transmission delay makes cloud-hosted AI inference impractical, requiring TinyML instead of an IoT solution. This involves a review of real-world applications that rely on edge computing solutions involving on-device inference. Another objective is to find information on simulation environments used for testing to provide ideas or models for the thesis research simulation environment. The scope of the research is inference, not training. For a real-world TinyML use case involving a commercial product, training is likely to occur in a centralized location, but inference must be performed locally from within each device.

The remainder of this paper is organized as follows. Section 2 provides background information on TinyML, including its measurement versus IoT solutions, the ML algorithms used, the hardware used, and the performance metrics employed for assessing the experiments. Section 3 explains the research methodology for this literature review, including the research questions, study selection criteria, data extraction process, and results. Section 4 presents the findings, Section 5 discusses the results, and Section 6 presents the conclusions.

## 2. Background

The introduction provides a brief overview of TinyML and this review’s objectives. This section gives a detailed description of TinyML, including its relationship to IoT, hardware used for TinyML implementations, software and machine learning techniques, and metrics used for performance assessment of TinyML implementation experiments. Table 1 lists several state-of-the-art studies on TinyML, its architecture, and use cases.

### 2.1. TinyML vs. IoT

Traditional IoT architectures rely heavily on a multilayered approach for data processing and inference. Surveys on the structure of IoT systems describe a hierarchical structure consisting of the perception, network/transport, middleware/processing, and application layers. This architecture relies on data movement between layers, with sensor data traveling from edge devices through network nodes before reaching the cloud servers for processing. This cloud-based approach offers significant computational resources but has inherent latency and bandwidth constraints that can impact real-time applications. Even with the introduction of intermediate processing layers such as fog computing and mobile edge computing, IoT architectures still face fundamental limitations in scenarios requiring immediate response times. IoT solutions carry risks regarding data privacy and security, as data packets can be intercepted when moving to and from the cloud via man-in-the-middle and replay attacks [4].

TinyML presents a fundamentally different architectural approach by integrating machine learning capabilities directly within edge devices. TinyML enables ML tasks to be executed on microcontroller units with severe resource constraints, typically operating with less than 1 MB of flash memory and with limited computational capabilities. This architectural shift dramatically reduces the dependency on network connectivity and external processing resources. The performance implications of this approach are significant. Whereas cloud-based IoT solutions typically exhibit latencies in the range of 10–500 ms, TinyML solutions can achieve latencies as low as 0–5 ms for inference tasks, with only a slight decrease in accuracy from 95% to 85% in comparative analysis [1].

The tradeoffs between these architectural approaches are prominent in performance-critical applications. Comparative surveys found that TinyML’s local processing capability provides several key advantages, including enhanced privacy through data localization, reduced bandwidth requirements, and improved reliability in environments with limited connectivity, but with constraints on model complexity and accuracy. Applications requiring complex model architectures or frequent model updates may still benefit from cloud-based processing, whereas time-sensitive applications with well-defined inference tasks are often better served by TinyML implementations. Some implementations involve hybrid solutions that attempt to use the advantages of both approaches and balance the disadvantages, at the risk of increasing complexity in system design and solution management [1].

Edge inference can be performed on the device, from an external server, or a combination of both. On-device inference executes ML inference on the same device that contains the sensors tasked with collecting data and software that extracts features from the data. This approach ensures minimal data transmission latency and lower risk of data leakage. The main drawback of hosting these processes on a device is that large, complex ML models cannot be run on-device due to the computational power and memory constraints of edge devices. This is known as a computational bottleneck, and some edge AI configurations attempt to alleviate it by moving some or all inference tasks to an external server, particularly to run larger models that are too large to run directly on the edge. This method requires edge sensors to constantly transmit data to and receive inference output from the server, which increases bandwidth requirements and results in latency constrained by the data transmission speed. Different configurations are designed with the inference task in mind; a computationally intensive task requires a powerful server with greater computational capacity, whereas an environment with weak inter-device communication or a task that requires near-instant transmission between the sensor and model would encourage inference from the edge device [5]. Some edge ML configurations place the sensors and the feature extraction part of the model on the edge device with the rest of the model remaining on the cloud. This is known as cooperative inference and results in a lower data transmission volume because the edge device sends only the feature vectors to the server rather than the whole sensor data. The more computationally and memory-intensive portion of the model carries out inference from the server, allowing for more intelligent and powerful models to run. Carrying out feature extraction on the device also reduces the communication overhead compared to carrying it out on the server, although privacy risks remain due to potential transmission data leaks. As edge inference is task-oriented, the degree of on-device vs. on-server computation varies depending on the task and environment [5].

Given low-latency constraints (0.3 s or less), on-device inference is comparable to on-server inference and cooperative inference in terms of accuracy and power use. As latency constraints become looser and reach 1.0 s, on-server and cooperative inference shows more power efficiency. This suggests a general latency range (below 0.3) where on-device inference can outperform other types [5].

### 2.2. Machine Learning Algorithms in TinyML

Machine learning algorithms deployed in TinyML environments require significant adaptations owing to the resource constraints. These adaptations often focus on three key techniques: knowledge distillation, pruning, and quantization. Knowledge distillation is a framework for transferring knowledge from a large pretrained model to a smaller model and enables the compression of complex neural networks into forms suitable for microcontroller deployment. This process involves training a compact network to mimic the behavior of a high-capacity network while maintaining acceptable accuracy levels, although typically with some performance tradeoffs [4].

The optimization of ML algorithms for TinyML deployment extends beyond simple model compression. Studies on model optimization show that techniques such as pruning can reduce the network size by up to 13 times without significant accuracy loss by systematically removing less important connections and neurons. This is performed iteratively, with networks undergoing multiple rounds of pruning and retraining to maintain performance. Quantization serves as another critical optimization technique, reducing the precision of weights and activations from 32-bit or 64-bit floating-point numbers to 8-bit or lower fixed-point representations, thereby enabling significant memory savings while preserving most of the model’s accuracy [1].

These algorithmic adaptations make machine learning algorithms more practical for real-world TinyML applications. This is because traditional deep learning models require hundreds of megabytes of memory and substantial computational resources, making them impractical for direct deployment on microcontrollers. Using these optimization techniques, TinyML algorithms can achieve compression ratios of up to 49× while maintaining acceptable accuracy levels [1]. Other surveys provide examples where optimized models reduced their memory footprint more than 90% while preserving most of their inferential capabilities. These significant reductions in resource requirements render previously impractical applications viable for edge devices. The ML model structure pre- and postprocessing was individually designed to account for varying sensor input data formats [6].

### 2.3. TinyML Hardware

TinyML algorithms are run on microcontroller units (MCUs) along with accelerators to assist with ML inference. MCUs designed for TinyML applications typically operate with severe constraints; most devices have clock frequencies ranging from 100 MHz to 480 MHz, flash memory capacities under 1 MB, and SRAM sizes between 128 KB and 512 KB. These limitations fundamentally shape how machine learning models can be implemented and executed. ARM Cortex-M series processors have become central to TinyML deployments, with Cortex-M4 emerging as a popular choice because of its balance of processing capability and power efficiency [1].

The TinyML hardware ecosystem is marked by significant heterogeneity, with many types of MCUs used and low standardization [6]. Device specifications depend on the use case and contain a variety of sensors, memory sizes, and runtimes.

Specialized hardware accelerators are also used to improve the performance of neural network computations within the constraints of edge devices and to assist with on-device machine learning inference. Hardware platforms such as Google Edge TPU and specialized neural processing units (NPUs) are used to execute matrix multiplications and other common ML operations more efficiently than general-purpose MCUs. These accelerators can achieve performance improvements of up to 20 times compared to standard MCU implementations while maintaining power consumption within acceptable limits for battery-powered devices.

Power efficiency is a critical factor in TinyML hardware selection and design. Surveys find that most TinyML applications target devices that must operate on battery power for extended periods, often months or years [1]. This requires sophisticated power management techniques and specialized hardware architectures. TinyML-optimized MCUs can achieve inference operations while using only 25–300 mW of power, which allows for the development of always-on applications that were previously impractical with cloud-based or traditional edge computing approaches.

### 2.4. Performance Metrics for TinyML Experiments

The evaluation of TinyML systems relies on a set of performance metrics tailored to both the hardware and software requirements. The four most common metrics are the model accuracy, inference latency, memory utilization, and power consumption. These metrics are fundamentally interconnected in TinyML systems, with improvements in one dimension often requiring tradeoffs in others, as well as ideal solutions requiring an optimal balance between each metric to accomplish their intended goals. As a result, system evaluation must be holistic and consider all tradeoffs, limitations, and use case specifications.

Latency measurements are a unique consideration when assessing TinyML solutions. Surveys of TinyML implementations show that they typically achieve inference latencies in the range of 0.18–300 ms, which is significantly lower than the 10–500 ms commonly observed in cloud-based solutions [1]. Latency measurements must account for not only the inference computational time but also delays in preprocessing steps and memory access operations. Survey analysis provides specific examples where TinyML solutions demonstrated latency reductions of up to 44.5× compared to traditional cloud-based approaches, although these improvements sometimes come at the cost of reduced model complexity and accuracy reductions [1].

TinyML assessments also showed that memory constraints can determine both model architecture and operational efficiency. Frameworks for evaluating memory usage consider both static memory requirements for model storage (typically limited to a few hundred kilobytes) and dynamic memory needed during inference operations. Surveys documented cases where optimized TinyML models achieved compression ratios enabling deployment on devices with as little as 256 KB of RAM while maintaining acceptable inference accuracy, representing a dramatic reduction from the traditional model requirements of hundreds of megabytes.

Power consumption metrics require careful consideration in TinyML evaluation frameworks. Power efficiency is often measured in terms of operations per watt, with successful TinyML implementations achieving significant improvements over more traditional approaches. The analysis shows that power consumption measurement must consider both active inference power and idle power states, as many TinyML applications require continuous operation on battery power. Surveys document typical power consumption ranges of 25–300 mW for TinyML implementations compared to 50–1000 W for cloud-based processing, showing that dramatic efficiency improvements are possible when optimized with on-device inference [1].

## 3. Research Methodology

This review was carried out using the guidelines for systematic literature reviews [7] and PRISMA guidelines [8]. The following subsections explain the research questions covered by the review; the information sources used; the eligibility criteria to assess, include, or exclude studies; and the methods used to collect data for the review.

### 3.1. Research Questions

This review covers the following research questions:

1. What is TinyML’s performance in a real-world low-latency application?
2. For which use cases could TinyML on-device inference prove optimal?

The first research question assesses TinyML’s performance, relying on the results metrics found in TinyML experiments, and the second research question combines performance observations with research gaps found in TinyML experiments to identify use cases where TinyML inference might prove superior to inference carried out from a cloud-hosted model. As a result, this literature review focuses primarily on the results and conclusions of the assessed studies.

### 3.2. Information Sources

As a starting point, we found recent TinyML literature reviews by searching in Google Scholar, IEEE Explore, and ACM Digital Library using the search string “TinyML Survey”, prioritizing the latest survey papers in the field. The six chosen surveys are reported in Table 1. Those six surveys were used to provide background information on TinyML and to identify several original experiments for the results section. Additional experiment sources were found in literature databases such as IEEE Explore and ACM Digital Library to cover aspects of TinyML implementation such as performance, latency constraints, and real-time edge AI implementations. The search results are explained further in Section 3.5.

### 3.3. Eligibility Criteria

Relevance: Studies must provide information to answer one or both research questions. For experiments, the studies must describe the ML frameworks and hardware used and the experiment’s use case. Experiments in which inference was not performed locally on a hardware device were rejected.

Language: Only studies written in English were used.

Publication Type: Only peer-reviewed journals and conference papers from databases such as IEEE Explore and ACM Digital Library, and references cited within those papers, were included.

Repetition: Only newer surveys whose results and conclusions were not previously covered were included. Older surveys were considered out of date and excluded.

On-Device Inference: Chosen experiments had to carry out inference from an embedded device to be considered as TinyML. Studies that used external cloud-based or server-hosted inference models were excluded. 

Usability: Experiments that proposed a machine learning framework without also including a use case example were excluded. 

### 3.4. Data Collection

Many citations in the TinyML surveys were older reviews of TinyML, smaller and less comprehensive surveys, or older papers describing research challenges and future ideas for the field. These studies were identified by their titles and abstracts containing phrases such as “A systematic review”, “A comprehensive survey”, “Current progress, research challenges, and future roadmap”, and ”opportunities and challenges”. These studies did not provide experimental results, and many cited each other. The literature search prioritized choosing wider-breadth and newer surveys and assumed that such surveys provided sufficient information that the smaller and older surveys cited could be ignored as a result. Table 2 lists examples of rejected surveys.

Many experiments have been conducted using a remote device such as a cloud server to perform inference on input data. Examples of the experiments rejected on those grounds are listed in Table 3.

Some TinyML studies proposed new models and architectures to carry out TinyML operations but did not offer any real-world use case for the experimental environment. Instead, these studies described a hypothetical model that could implement an architecture and tested their model for performance in a generic setting rather than measuring its performance for a specific use case. Examples of studies rejected on these grounds are listed in Table 4.

### 3.5. Results

Overall, 22 papers on TinyML experiments that met the eligibility criteria described in Section 3.3 were chosen for analysis, which were taken from citations of the six comprehensive surveys examined. Of the 22 studies, seven of these papers covered the field of healthcare, nine additional papers covered the field of ecology, four papers covered the field of vehicular object detection, and two papers covered other miscellaneous use cases. Table 5 lists all survey-sourced experimental studies.

An additional four experiments were taken from searching specifically for low-latency experiments using TinyML or Edge AI and excluding those that used the same use case. The search string was entered into Google Scholar, and consisted of the following string:

(“instantaneous inference” OR “real-time”) (TinyML OR “Edge AI”) “implement”—review

This string returned 2150 studies, which were filtered to 1880 studies by limiting the results to studies released since 2021. The first ten pages consisting of the top 100 hits were scanned. Table 6 lists the four studies chosen from the results.

**Figure 2:**
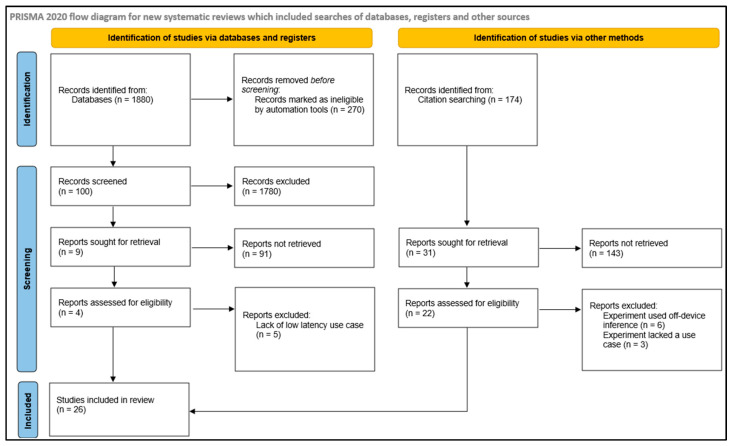
PRISMA selection flowchart.

Figure 2 shows a PRISMA flowchart of the search process, including the selection of references from surveys and experiments.

This search process provided 26 experimental studies to assess for their results. The results are further described in the following section.

## 4. Findings

This section explains the results of TinyML for use in various application categories extracted from the chosen experimental studies. Data from demonstrations of TinyML use cases, and in particular, the reasons the authors gave for their choice to use a TinyML solution over an IoT or cloud-based solution in their experiments were examined. Their results and conclusions were analyzed to assess how these experiments fit TinyML’s application history.

The data extracted from all experiments include the following:

(1) The proposed use case of the experiment.
(2) The reason TinyML inference was chosen over IoT or cloud inference.
(3) The type of hardware and NN software used for the experiment.
(4) The results of the TinyML experiment.

### 4.1. TinyML Applications in Healthcare

This subsection covers TinyML experiments related to the field of healthcare. Most of these studies have covered patient diagnostics and health monitoring. Table 7 lists the results of the healthcare application experiments.

Experimental studies on TinyML implementations in healthcare have focused on data processing assistance for various treatments or detection procedures. One experiment involved testing TinyML as a solution to enhance speech for use in hearing aids [26]. The experiment used an STM32 microcontroller unit to represent the hardware constraints of a hearing aid as a model for their experiment. Their model’s memory size was pruned by 47% using experimental pruning techniques to optimize the model and fit it onto the device. The experiment’s survey participants expressed a moderate preference for the enhanced audio sample over the unprocessed audio sample, and the computational latency was sufficiently reduced to 4.26 ms—below the benchmark requirement of 10 ms. Their experiment found the optimized model satisfactory and a latency low enough to consider pruned RNNs for their task, and the TinyML implementation allowed for audio processing on the edge device without relying on real-time IoT service.

Other TinyML experiments cover medical detection scenarios, with one example being the implementation of a TinyML model for detecting gait deficit among patients with Parkinson’s disease [27]. The researchers assumed that latency and network connection dependency were the reasons for choosing TinyML over the other solutions. The embedded model was run on an ATMega2560 microcontroller. Their results suggested that these techniques worked to accurately classify Parkinsons symptoms and could be extended in future work to carry out predictive analysis. They concluded that their hardware was appropriate for the experimental tasks and suggested that the experiment could also be scaled further to a wider range of embedded devices. 

Another detection experiment analyzed electrocardiogram results using a TinyML solution to detect and classify cardiac arrhythmias [28]. The experiment used a Chip nRF52 from Nordic Semiconductor with an ARM CortexM4 processor as the hardware base to run the model. The inference model was a CNN running a CMSIS-NN library designed for ARM Cortex-based processors. The experimental results showed a model accuracy of 87% on the testing set, a memory requirement of 210 KB output binary to run the network, a latency execution time of 95 ms, and a power usage of 21 mW/h. For future work, they suggested comparing their inference library with other libraries such as TensorFlow Lite for Microcontrollers (TFLM) to find the best performing ML architecture solution for their experimental task. 

TinyML has also been shown to be able to detect medical problems from visual data as well. One such experiment used a TinyML model for the detection and classification of liver lesions using visual data fed into the model [30]. The experiment did not use a microcontroller or hardware device for hardware but instead used an automated CAD hardware model fitted for use in a TinyML environment with small-memory constraints. The model consisted of a deep neural network (DNN) architecture with pretrained weights from Keras Applications, with an Adam training optimizer for maximum accuracy. The experimental results showed an 80% accuracy in detecting liver lesions for experimental data in a model with a 75 ms delay. These results were satisfactory enough to support the use of TinyML inference for acute detection of lesions.

Two additional experiments investigated the use of TinyML for seizure detection in patients with epilepsy using different neural network models running on different hardware devices. One group of researchers ran their experiment on an STM32L476 ARM Cortex microcontroller, with detection processed on the device [29]. The study results showed high accuracy rates of over 90%, and a low false-alarm rate. The other seizure detection experiment was performed using a BioWolf wearable ExG device with a multicore processor, with the seizure detection model implemented on the device [31]. Both experiments measured detection success using the Recall Score to measure accuracy, because their use cases involved both feature detection and classification. Both studies used Random Forest as a major classification algorithm, finding it the most suitable for classification on a resource-constrained platform.

One study focused on detecting colorectal cancer polyps in patients [46]. They chose to embed their model on a hardware device to reduce the power consumption and increase the accuracy. They used a CNN model for classification and implemented it on a medical capsule robot (MCR) modified to contain an MCU with a four-layer CNN implemented on the MCU. The images captured by the MCR sensor were compressed and sent to the model, which classified them. Their model was able to successfully classify cancerous polyps on the test dataset with an accuracy of 92.8% while maintaining low power use, using only 2.5488 mW at a clock speed frequency of 8 MHz, with an inference latency of 1.6 s. They concluded that their four-layer model had a slightly lower accuracy than other common CNNs but that the power and memory efficiency make it usable for classification tasks.

Another study used TinyML to detect respiratory conditions from sound recordings, distinguishing between healthy lung respiration and those from asthma patients [49]. The researchers chose on-device inference to reduce diagnosis result latency and enhance data privacy by avoiding transmission across a network. The hardware used was an Arduino Nano MCU with an ARM Cortex processor containing 256 KB of RAM. The inference was done with a custom-designed CNN trained using TensorFlow Lite Micro. The custom CNN model returned a 96% accuracy rate and a 97% precision and recall rate on the test dataset, using only 12 KB of RAM for inference, approximately 250 KB of flash storage for the model, and with an inference time of 127 ms.

Overall, TinyML’s use in healthcare applications mainly focus on diagnosis, with ML models chosen for classification purposes. The hardware used is typically specialized microcontrollers and wearable devices, particularly the ARM Cortex series. The key reason for choosing TinyML is resource constraints, in particular memory and power constraints. Other common reasons include system security and data privacy, both of which were hypothesized to be more secure with on-device inference computing compared to cloud-based IoT. TinyML inference latency remained around 100 ms with complex diagnostics such as cancer lesions taking 1 to 2 s on average. Inference accuracy rates ranged between 80 and 99%.

### 4.2. TinyML Applications in Ecology

This subsection covers the TinyML experiments related to ecology, including agriculture, water treatment, and environmental monitoring. Table 8 lists the results and implications of the experiments.

Research into TinyML applications in smart farming has found that the majority of TinyML solutions deal with crop management, such as moisture and temperature data processing, irrigation optimization, and yield efficiency optimization [1]. These experiments chose TinyML over IoT because of issues with latency and security that prevent the effective use of cloud-based inference solutions. Machine learning techniques in smart agriculture often rely on processing data collected from sensors to analyze crop status and detect the presence of disease or pests [41]. This processing is often performed under time, power, and memory constraints, as accurate results must be delivered within time to ensure a successful response, and detection must be cost-effective and deployable at scale and across diverse ecological environments and agricultural settings.

One such experiment used TinyML to forecast temperature in greenhouses using sensor data to predict temperature patterns [33]. Their neural network model was run on an Arduino Nano 33 BLE Sense for greenhouse monitoring. This platform proved sufficient for running their neural network models while consuming very low power compared to traditional computing platforms. The resulting experimental model used only 0.17 W of power compared to the base model which used 3.5 W, suggesting that temperature forecasting can be implemented successfully on the edge even on extremely power-constrained devices.

TinyML for edge audio processing has also been tested, with a trained model deployed on an Arduino Nano connected to a 0.9-inch display put to test [38]. The results showed that mosquito wingbeats can be recognized by a model collecting data from Arduino. Limitations include distance issues, as audio quality is dependent on the sample distance from the device, and the testing environment had certain sound constraints. The model exhibited an accuracy of 88.3% on a testing dataset of mosquito wingbeat samples. The inference time was 337 ms, with a RAM consumption of 9.2 kB and a flash usage of 43.4 kB for one second of data.

TinyML image classification can be carried out on an OpenMV Cam STM32H7 Plus, with an LR-Net model embedded in the camera’s ROM. The resulting TinyML system can process and classify 15 images per second at an accuracy of 98.0%, with the model using only 13 kB of the 31 kB camera RAM space [40]. STM32CubeAI was used to generate a C code file from the neural network model to perform inferences from the camera hardware.

Other classification experiments focused on using embedded models to detect and classify gases [42]. One implementation had a classification accuracy of 72% for detecting gases with a sensor and classifying them into one of four categories (Ammonia, Methane, Nitrous Oxide, or neither) The main proposed use case of this experiment is to assist farmers in monitoring air quality on an edge probe with less need to remain connected to the cloud. The researchers aimed to develop a low-cost, low-power probe capable of measuring the presence of environmentally harmful gases. The experiment used TinyML inference over IoT to increase transmission efficiency and avoid cloud-to-device traffic. The hardware included several sensors connected to an STM32 MCU running a pretrained ANN converted from Python to C code using XCubeAI. The resulting accuracy was calculated by comparing the predicted values to the true values and found five classification errors in 18 test patterns.

TinyML solutions have also been tested for vehicular emission detection to monitor CO_2_ emissions from vehicles using engine sensor data. The experiment used a Freematics ONE+ hardware platform featuring an ESP32 microcontroller for vehicular emissions monitoring [32]. TinyML was chosen over IoT inference to reduce power costs and reliance on network infrastructure and enable real-time, distributed monitoring near pollution sources. The system successfully processed OBD-II sensor data while maintaining low power consumption. They implemented unsupervised TinyML with Typicality and Eccentricity Data Analytics (TEDA) to process vehicular emission data. The experimental results for vehicle emission detection showed 94% accuracy using TEDA, with inference times of approximately 1 ms and RAM usage of only 1.5 KB. 

Another TinyML experiment was conducted to carry out environmental predictions and atmospheric pressure forecasting and to determine whether a neural network running on an MCU can reliably predict weather patterns [34]. The experiment used Long Short-Term Memory (LSTM) networks to predict the atmospheric pressure. They used two LSTM cells with 30 units per cell and dropout layers at a 20% drop rate. The hardware used an STM32F401RET6 microcontroller with 512 KB of FLASH memory and 96 KB of SRAM. The prediction results had a Root Mean Square Error of 0.0255. Their system demonstrated the successful operation of deep tiny neural networks running on an MCU with memory constraints.

Another experiment examined the use of TinyML to detect cholera contamination in communal tap waters in rural areas. The researchers used TinyML to process physicochemical parameters of water to predict water-borne cholera presence, assuming that traditional laboratory testing methods are expensive and impractical for deployment in rural Africa [35]. The implementation used an embedded kit that could carry out offline inference, which included an ARM Cortex M4 processor. Their model used Support Vector Machine (SVM) as the primary ML algorithm, which was specifically chosen for its effectiveness with small datasets and nonlinear pattern recognition. They used model compression to reduce the size to fit on the hardware for the model to run. The experimental solution had an accuracy of 94% for SVM classification on the testing dataset, with an output latency of 1 ms, a memory usage of 1.6 KB for RAM and 15 KB for flash, and within the power constraints.

Model sizes below 140 kB can be used to successfully predict plant growth status and disease presence with 96% accuracy on a 10-class dataset. Such models are compact enough to fit within a battery-powered Sony camera system and require a low power rate of about 2.63 mW per hour to operate, allowing for easy use and deployment in an agricultural environment bound by power constraints [41].

One experiment integrated TinyML with unmanned aerial vehicles (UAVs) for smart farming. On-device inference was chosen over cloud inference to reduce bandwidth requirements, enhance privacy, and reduce energy consumption. The model predicted soil moisture content using a DNN and LSTM model on an ESP32 microcontroller running TFLM. The resulting DNN achieved a 97% accuracy rate with an average inference time of 97.8 ms. The experiment found that DNN models with smaller LSTM structures require less memory to infer and could infer faster than those with larger LSTM structures [47].

Overall, the use of TinyML for ecology includes a wide breadth of applications ranging from sensor data processing to audio and image classification. As a result, a wide range of neural networks are used specific for each application. Resource constraints include memory, power, and communication bandwidth, with the latter resource dependent on the local infrastructure of the application environment. Inference latency ranged from 0.1 to 1 s, with accuracy ranging from 98% for simple image classification to as low as 72% for complex gas analysis. These results show that TinyML’s performance in ecological experiments is varied and dependent on the task complexity and the experiment setting.

### 4.3. TinyML Applications in Vehicular Detection

Another field for TinyML experiments includes vehicular assistance software, particularly related to sensors and object detection. Table 9 lists the related experiments, their results, and the implications.

Intelligent cars offer a new platform for the development of embedded vehicle service applications [43]. TinyML implementations in vehicles stem from the existing research gaps identified in IoT-based intelligent vehicle implementations. IoT solutions rely on direct data-streaming connections between a vehicle processor and a centralized cloud server. This current arrangement suffers from scaling issues where the cloud server may not be capable of managing a high volume of data transmission, reception, and processing beyond a certain scale [43]. Solving this problem requires localizing certain ML system components such as inference onto the edge computing layer to limit the volume of data that would have to be streamed to and from the cloud. This research gap offers opportunities to experiment with the use of edge inference for intelligent vehicles, both in terms of applying such a model to different use cases and optimizing the degree of localization between devices and the cloud.

Use cases for TinyML systems in intelligent vehicles often involve the detection of other road objects such as vehicles or pedestrians [36] and the detection of road quality issues such as potholes. TinyML experiments included models for real-time vehicle and pedestrian detection (VaPD) in automotive applications for use in driver assistance systems. One such experiment tested whether TinyML could process camera input data and detect both vehicles and pedestrians within model size constraints [36]. They implemented a vehicle and pedestrian detection system on a Raspberry Pi 4 and an NVIDIA Jetson Nano 2 GB. The Raspberry Pi 4 was enhanced with a Coral USB Accelerator with an Edge TPU coprocessor for optimal inference capability. The edge model uses a Tiny YOLO v3 architecture with Tucker tensor decomposition as an optimizer to decompose the convolutional layers and reduce the parameter count. This technique was chosen specifically to join decomposition and fine-tuning in a single step as part of the optimization process. The results achieved an experimental precision of 77.5% with their optimized model, using a much smaller memory size (22 to 32%) compared to their baseline model, and a storage cost of 10.7 MB compared to an 875 MB solution. The experimental results showed that functional object detection on an edge vehicle can be successfully implemented even with a smaller model size and storage constraints, allowing for more memory-efficient implementations.

Detection algorithms also include vehicle drivers, as shown in one experiment [39]. The experiment used TinyML to detect signs of driver drowsiness for a vehicle safety assistant system. The experiment selected lightweight sensors with a locally run embedded model to minimize computational cost and reduce inference latency as much as possible, as cloud-hosted inference was considered to have an unacceptable latency delay. The main challenge identified was the high training cost for DL models, both in terms of computing power and training data, to make accurate predictions. The experiment used several lightweight DL models, quantized to reduce their size, to perform inference. The experiment aimed to cover existing the limitations they found in driver detection experiments, namely, issues with accurately identifying different driver head movements and issues with user-unfriendly means of driver detection. The resulting model had an accuracy of 0.9964.

Another similar experiment examined TinyML for real-time bus passenger detection and counting for smart public transport systems [37]. The experiment involved optimizing a Tiny YOLO network to accurately detect passengers while still meeting resource constraints. This involved a model adjusted to use a depth wise decomposition, carrying out decomposition and fine tuning in separate steps. They used batch normalization and LeakyReLu as activation functions to reduce the computational complexity as much as possible. The experiment results had a detection accuracy of 0.945 during rush hour, with the file size decreasing from 60.5 MB to 7 MB compared to the base model.

When implemented on a camera sensor in an intelligent vehicle, a TinyML system can detect road anomalies such as speed bumps and potholes at a high rate, maintaining an F1 score of 0.76–0.78 using multiple iterations of an experimental driving route of an intelligent vehicle [43]. The study did not compare memory or power use, which may provide an additional topic of research when comparing the efficiency of intelligent vehicle TinyML implementations rather than just their accuracy score.

The use of TinyML for vehicular detection mainly includes image detection and classification of obstacles, pedestrians, passengers, and vehicles. These applications favor TinyML solutions due to a need to overcome bandwidth latency delays and avoid communication link problems. The results depend on the setting and available resources, with accuracy ranging around 76–78%, indicating a need for improvement.

### 4.4. Other TinyML Applications

TinyML is also used for other applications such as industrial design, edge device security, and edge model deployment. Table 10 lists studies that used TinyML for applications other than healthcare, ecology, or vehicular detection.

Other experiments have focused on the application of TinyML to machine design and production. The experiment aimed to use TinyML to perform efficient real-time fault detection of operating machinery to reduce machinery production and maintenance costs. The TinyML model was tasked with monitoring the condition of industrial assets and detecting anomalies therein. Data acquisition, training, and inference were performed on an edge device. The framework uses an MCU attached to an accelerometer to detect vibration signals. The TinyML system achieved an anomaly detection accuracy of 99.9% when tested on a centrifugal pump [44].

The heterogeneity of TinyML systems, particularly hardware, provides barriers to their widespread use in industrial settings where standardization and scale are paramount [6]. Insufficiently detailed documentation of the TinyML model distributions provides barriers to their integration in large-scale industrial systems. The privacy, latency, and energy efficiency benefits of TinyML still draw demand for its use in industrial settings.

TinyML was used over IoT to avoid a bottleneck problem with transferring and receiving data caused by bandwidth constraints and server response time causing inference delays. This problem was hypothesized to worsen as the number of machines increased. The experiment used edge training as well as inference, as they believed that their anomaly detector must train the device on the same device on which inference is performed. The experiment used a STM32 MCU with 1 MB of RAM and 2 MB of flash memory, and the core was an ARM Cortex. The system used a signal processing technique called Wavelet Packet Decomposition to reduce the data dimensions to fit the MCU’s memory constraints. Input data were separated into low- and high-frequency regions. The autoencoder was trained in C by using a backpropagation algorithm. The network was trained sequentially to account for data storage limits and functioned by comparing the Mean Square Error (MSE) of the sensor data to a set anomaly level. If the error exceeded the anomaly level, a signal was sent to investigate the machine. The system output was evaluated by comparing the ratio of True Positives, True Negatives, False Positives, and False Negatives to assess the output accuracy. The study claimed a training model accuracy of 0.997, with only one false positive out of 3400 samples, and a testing accuracy of 1 for 100 test samples. The main limitation is the test sample count, as 100 tests on only one machine may be insufficient for this use case. The test results are also insufficiently explained, as no diagram is provided to analyze them; the results (accuracy, precision, and F1 score) are only mentioned in one line.

One experiment deployed and tested ML models on an integrated circuit known as an FPGA. An edge inference solution was used to reduce the classification latency, because the task of detecting and classifying malware in real time was very time sensitive and required a detection speed measured in microseconds [45]. The FPGA was loaded with software to detect and classify malware and side-channel attacks from hardware register data collected from the device. As the goal of the experiment was to design and test a detection system for hardware-level malware, they chose an edge AI implementation to perform detection and classification on an embedded system. Multiple model types were tested on the same hardware device to compare their performance and assess their suitability. The experimental results showed varying degrees of accuracy for the different implemented models, with the J48 model displaying the highest F1 score of 0.918 for malware detection among the tested models.

The executed ML training pipeline returned a trained model and a set of evaluation metrics that were deployed via an automated deployment pipeline that ran immediately after validation. The model deployment script was carried out within a Docker service image, which deployed the model on the edge device where it could carry out inference on real-time data generated by the edge device’s sensors. The experiment simulated a real use case and applied their ML pipeline against it. The resulting ML pipeline successfully trained their model on the cloud and deployed it to an edge device. The deployed model then carried out a principal component analysis against humidity and temperature measurement data collected in real time by the device sensors. The training cloud server had four cores and 16 GB RAM, and the edge device was a Kunbus, which is similar to a Raspberry Pi Core. The ML model was run inside a Docker container in the edge device and required 1.267 s to run. Building the docker image from the trained model took 457.4 s and the combined deployment steps took approximately 38 s. The running model could process more than 12,000 requests per minute and successfully predicted all anomalies in the test dataset correctly [51].

## 5. Discussion and Future Work

In the following subsection the results of experimental studies that use TinyML are discussed to provide an answer to the first research question and to analyze its implications for the use of TinyML inference. Existing research gaps found in the cited studies are covered, along with any new gaps identified in this literature review.

### 5.1. Evaluation and Implications

Experimental studies of TinyML implementations show sufficient ability to carry out complex machine learning tasks with high accuracy and within stated resource constraints. These experiments demonstrate usability across multiple fields and the versatility of the hardware and model structures, designed according to the experimental environment needs, shows that TinyML can be implemented across a wide range of design specifications, further proving its versatility as an alternative solution to IoT. The experimental results show that TinyML can bridge the gap between the computational requirements of modern machine learning models and the constraints of embedded devices. The successful implementation of complex tasks such as medical diagnosis, environmental monitoring, and real-time object detection within strict power and memory constraints shows that TinyML is a viable solution for deploying AI capabilities in resource-constrained environments.

The consistently low inference latency may allow TinyML to provide solutions that IoT inference cannot, especially for use cases where extremely low delay requirements prohibit the use of cloud-based data transmission, such as for autonomous vehicles and healthcare data monitoring. Alternatively, environments where data transmission is difficult, such as remote or underdeveloped areas, may also provide an environment where TinyML is the only workable edge computing implementation.

The memory and power efficiency of TinyML solutions also shows viability for widespread deployment on small battery-powered devices. Many of the experiments in this study attained accurate results with highly compressed models below 1 MB in size, which enables the use of machine learning techniques on small, embedded devices.

For healthcare applications, TinyML experimental results demonstrate high detection accuracy for ailments such as seizures, cardiac arrhythmias, and lesions. Additional benefits of TinyML detection include superior data privacy and a lower reliance on bandwidth networks to transmit sensitive health information between the data-collecting sensor and the processing model.

Many of the experiments found model accuracy highly dependent on input data quality. This includes poor processing of data, biased datasets, and incomplete data gathered by low quality sensors. This phenomenon can create a potential bottleneck that limits the usefulness of sophisticated ML models by the quality of the sensors attached to the device. It also restricts targeted analysis of embedded ML models due to the sensor’s role in inference output quality.

The experiments also found that model size reductions caused by optimization techniques also led to a reduction in model complexity. This may reduce the scope of problems that TinyML can address compared to cloud-based solutions with fewer resource constraints. This might be addressed in future studies that further investigate the relationship between complexity, model size, and model task depth to pinpoint which tasks require model storage capacity beyond what is available on an embedded device.

### 5.2. Limitations and Bias

This review primarily focuses on studies cited within comprehensive surveys and found via search strings, potentially missing relevant research found in other types of publications such as conference papers. The review’s exclusion criteria regarding studies that use external inference may also have eliminated comparative analyses that would provide context for TinyML implementation choice.

The evaluation metrics extracted from TinyML experiments were not standardized across experiments. Accuracy metrics were reported as detection precision, F1 scores, and recall rates, making definitive comparisons about performance across domains more challenging.

Given that only successful experiments were included and analyzed, the reviewed literature may suffer from publication bias, as TinyML experiments with negative outcomes were not analyzed for their metrics or experiment settings. This may obscure significant challenges and limitations in TinyML implementation analysis.

These limitations may lead to an overestimation of TinyML implementation capabilities, as well as an underestimation of TinyML’s limitations, including deployment challenges, maintenance, and long-term performance stability.

### 5.3. Research Gaps and Future Work

One research gap could relate to dealing with disparities within TinyML systems between the sensors, model, and hardware capacity, as these three processes make up the core of edge inference systems. For example, a powerful model may still be limited by the quality of the data produced by the device sensors. Because the outputs of one portion become the inputs of the next portion, issues at any one stage (sensing, feature extraction, and inference) would compromise the quality of the final inference output. An insufficiently accurate sensor may result in missing information that compromise the accuracy of the output of a complicated inference task [5].

Given that TinyML experiments contain a diverse selection of hardware platforms, from Arduino Nano to specialized TPUs, one potential research topic could involve building standardizations framework for TinyML applications to accelerate adoption and integration of TinyML into larger networks. Further studies can also investigate other means of scaling TinyML setups for large deployments involving thousands or millions of devices. Other methods to help address standardization and deployment challenges include better benchmarking, including frameworks specific to TinyML, more comprehensive TinyML model deployment pipelines, and standards for API middleware to enable better hardware–software coordination for TinyML applications, enabling TinyML developers to focus on application development rather than hardware and software environment design.

Another topic of interest could be investigating TinyML model training and model architectures specific to resource-constrained embedded networks. Current implementations often involve building and training the model on a large cloud server and then compressing it via pruning to fit the model onto a small device. Devising a new system that builds and trains the model on an architecture specific to embedded devices and then testing that model against a compressed model could contribute to the design of more capable models. The gap between resource-intensive training and efficient inference could also be investigated, as more efficient model training could improve the scalability of TinyML edge networks. These advances could incorporate new innovations in model training such as continuous learning, federated learning, and transfer learning.

The most common reasons to choose TinyML over IoT in the analyzed experiments were inference latency reduction and data privacy. Bandwidth was a major concern for researchers, especially for higher volumes of data traffic between the sensors and the cloud. Each new cloud-device connection offers a new point of attack for a malicious actor, as data can be intercepted and altered during transmission. New innovations such as smart cities, integration of wearable devices, and public transportation networks only increase the scale of potential privacy risks, network lag, and bandwidth strain. TinyML has potential to assist in the scalability of edge computing, which can be explored further in future research.

## 6. Conclusions

This review has examined the current state of TinyML as a viable alternative to cloud-based inference for applications where low latency and bandwidth efficiency are critical and make traditional IoT solutions impractical. By analyzing experimental studies in the fields of healthcare, ecology, vehicular detection, and industrial settings, we have identified key trends in TinyML research and the significant potential and remaining challenges in TinyML implementations.

TinyML demonstrates compelling advantages in scenarios requiring rapid, secure, and private inference in environments with limited connectivity. The successful implementation of complex machine learning algorithms within the resource constraints of small, embedded devices demonstrates that edge artificial intelligence has matured beyond promise to a level allowing practical implementation.

Despite these significant advances, some research gaps remain in areas such as scalability, standardization, model optimization, energy efficiency, and security. The heterogeneity of current TinyML implementations shows its depth of versatility but also provides challenges for widespread adoption and deployment at scale. Addressing these gaps is essential for advancing the deployment of TinyML across future application domains.

The findings from this review suggest that TinyML may become an integral part of next-generation IoT systems, enabling a new class of intelligent devices that operate effectively at the network edge. Future research should focus on refining the TinyML frameworks, improving interoperability with existing IoT ecosystems, and exploring novel applications that push the boundaries of edge intelligence. As TinyML continues to evolve, its role in enabling real-time, resource-efficient AI solutions is likely to expand, shaping the future of intelligent edge computing.

## Figures and Tables

**Table 1:** Surveys cited in Section 1 and Section 2.

Survey Reference	Year	Overview	Limitations
Abadade, Y. [1]	2023	This survey provides a comprehensive analysis of TinyML, its architecture, and its use cases.	The study identifies current issues in TinyML but does not suggest how to solve them.
Capogrosso, L. [2]	2024	The survey describes all learning algorithms used in TinyML implementations.	The study insufficiently covers TinyML’s use cases.
Elhanashi, A. [3]	2024	The survey discusses TinyML’s use in various applications.	Their analysis does not provide a sufficient explanation of TinyML’s performance.
Tsoukas, V. [4]	2024	This survey classifies TinyML optimization techniques and reviews their applications.	The survey explains ML techniques and their applications in detail but offers limited breadth beyond that.
Liu, S. [5]	2024	This study explains TinyML edge inference and compares latency differences of edge vs. cloud inference.	More focused on edge inference rather than TinyML.
Ren, H. [6]	2022	This study provides a schema for managing TinyML models in IoT devices for industrial settings.	The study limits itself to industrial settings only.

**Table 2:** Rejected older or narrower breadth surveys.

Survey Reference	Year	Overview and Limitations
Han, H. [9]	2022	The study carries out a systematic review of TinyML and its applications and was rejected for being out of date.
Tsoukas, V. [10]	2021	The study describes how TinyML can be used in healthcare applications and was rejected for being old and covered by newer surveys.
Sanchez-Iborra, R. [11]	2020	The study reviews the use of TinyML in smart objects and was rejected for being out of date.
Ray, P.P. [12]	2022	The study is a state-of-the-art review of TinyML and its prospects and was rejected for being covered by newer surveys.
Dutta, D.L. [13]	2021	The study is a comprehensive survey comparing TinyML with IoT and was rejected for being covered by newer surveys.
Shafique, M. [14]	2021	The study reviews the state of research in TinyML and was rejected for being too old.
Greco, L. [15]	2020	This study covers edge computing in IoT and was rejected for being out of date.
Oufettoul, H. [16]	2024	The survey covers TinyML implementation tools and was rejected for being too limited in its explanation.

**Table 3:** Rejected experiments that used external inference.

Survey Reference	Year	Overview and Limitations
Giraldo, R. [17]	2020	The study uses a camera system to monitor crops and was rejected for using cloud-based inference.
Abdennadher, N. [18]	2021	This study devised a TinyML implementation to monitor electrocardiogram results for anomalies and was rejected for using cloud-based inference.
Matilla, M. [19]	2022	The study uses edge computing solutions to monitor crops and was rejected for using IoT inference.
Ihoume, I. [20]	2022	The study used a TinyML model to monitor greenhouse temperature and was rejected for using cloud inference.
Laureanti, R. [21]	2020	The study implemented emotion assessment in wearable devices using TinyML and was rejected for carrying out inference on an external PC rather than on device.
Ayata, D. [22]	2020	The study used TinyML to measure emotions in wearable devices and was rejected for using cloud inference.

**Table 4:** Studies rejected for lack of stated use case.

Survey Reference	Year	Overview and Limitations
Li, Z. [23]	2021	The study implements a CNN accelerator on an FPGA and was rejected for a lack of described experimental use case.
Krishna, A. [24]	2024	The study proposes and tests a new inference model for TinyML and was rejected for not including an experimental use case.
Sanchez-Iborra, R. [25]	2023	Proposes a cooperation framework between TinyML and edge computing but did not describe a use case for the framework.

**Table 5:** TinyML experiments chosen from surveys.

Study Reference	Year	Overview
Fedorov, I. [26]	2020	TinyML for speech enhancement in hearing aids
Gokul, H. [27]	2020	TinyML for detecting gait deficit in patients with Parkinsons
Faraone, A. [28]	2020	TinyML for detection and classification of cardiac arrhythmia using electrocardiogram results
Zanetti, R. [29]	2020	TinyML for detecting seizures in epilepsy patients
Caleanu, C. [30]	2021	TinyML for detection and classification of liver lesions
Ingolfsson, T. [31]	2021	TinyML for noninvasive monitoring of epilepsy patients
Andrade, P. [32]	2022	TinyML for CO_2_ emissions detection and monitoring in vehicles
Alati, M. [33]	2022	TinyML for temperature forecasting in greenhouses
Alongi, F. [34]	2020	TinyML for environmental predictions and atmospheric pressure forecasting
Ogore, M. [35]	2021	TinyML for detection of cholera contamination in communal tap water in Africa
Falaschetti, L. [36]	2024	TinyML for real-time vehicle and pedestrian detection in driver assistance software
Zhang, S. [37]	2020	TinyML for real-time bus passenger detection and counting
Trivedi, K. [38]	2021	TinyML for identification of deadly mosquitoes
Alajlan, N. [39]	2023	TinyML for vehicle driver drowsiness detection
Falaschetti, L. [40]	2021	TinyML for image detection of grape leaf diseases
Du, P. [41]	2022	TinyML for plant growth monitoring
Bruno, C. [42]	2021	TinyML for gas recognition in smart agriculture
Andrade, P. [43]	2021	TinyML for pavement anomaly detection for intelligent vehicles
Alireza, M. [44]	2021	TinyML for fault detection in rotating machines
Wang, H. [45]	2021	TinyML for edge device security
Lu, C. [46]	2024	TinyML for colorectal cancer polyp detection
Hayajneh, A. [47]	2024	TinyML for UAV assisted smart farming

**Table 6:** TinyML experiments chosen from search strings.

Study Reference	Year	Overview
Ren, H. [48]	2021	TinyML for event processing in industrial IoT
Abadade, Y. [49]	2024	TinyML for lung disease classification
Im, H. [50]	2024	TinyML for real-time vehicle intrusion detection
Bustamante, A. [51]	2023	TinyML for industrial edge deployment

**Table 7:** Experiments that covered TinyML in healthcare.

Study Reference	Year	Results and Implications
Fedorov, I. [26]	2020	The pruned model successfully processed audio samples on the hearing aid, with satisfactory quality and low transmission latency.
Gokul, H. [27]	2020	The TinyML MCU implementation could detect Parkinsons symptoms in patients.
Faraone, A. [28]	2020	The model could detect ECG irregularities with 87% accuracy under appropriate latency and power constraints.
Zanetti, R. [29]	2020	Over 90% accuracy in seizure detection.
Caleanu, C. [30]	2021	80% accuracy in liver lesion detection with a 75 ms inference delay.
Ingolfsson, T. [31]	2021	94–99% specificity for detection.
Lu, C. [46]	2024	92.8% classification accuracy on the test dataset, 2.5488 mW used per inference.
Abadade, Y. [49]	2024	97% precision and recall on the test dataset, 127 ms inference time.

**Table 8:** Experiments that covered TinyML for ecology.

Study Reference	Year	Results and Implications
Alati, M. [33]	2022	Greenhouse temperature forecasting successfully implemented under extremely low power constraints of 0.17 mW.
Andrade, P. [32]	2022	Vehicle emission detection accuracy rate of 94% within a 1 ms inference latency constraint.
Alongi, F. [34]	2020	RMSE of 0.0255 on an MCU-embedded model of 512 kB memory.
Ogore, M. [35]	2021	94% detection accuracy of cholera on an offline device within latency, model size, and power constraints.
Trivedi, K. [38]	2021	88.3% detection accuracy with a 337 ms delay.
Falaschetti, L. [40]	2021	98.0% classification accuracy at 64.2 ms per image inference time (15.5 fps), with ~13 kB ROM used.
Du, P. [41]	2022	Successful prediction of plant growth and disease status within a 136 kB model, model usable in battery-powered camera systems.
Bruno, C. [42]	2021	Embedded model has a 72% accuracy in detecting and classifying gas samples.
Hayajineh, A. [47]	2024	Validation accuracy of 99%, results found that fewer LSTM structures resulted in faster inference and less memory usage.

**Table 9:** Experiments that covered TinyML usage in vehicular detection.

Study Reference	Year	Results and Implications
Falaschetti, L. [36]	2024	Detection precision of pedestrians of ~77% on a model compressed to ~33% of its original size.
Zhang, S. [37]	2020	Bus passengers detected with high accuracy on a compressed model.
Andrade, P. [43]	2021	Speed bumps and potholes detected with F1 score of ~0.76; model deployed on an Arduino Nano.
Alajlan, N. [39]	2023	Quantized models were assessed for accuracy and model size. CNN model had greatest compression (0.05 MB), while the DRQ MobileNet-V2 model had the highest accuracy at 0.9964.

**Table 10:** TinyML experiments in other categories.

Study Reference	Year	Results and Implications
Alireza, M. [44]	2021	Model successfully trained on edge; test results show an accuracy of 1.
Wang, H. [45]	2021	J48 model embedded on a Pixel 4 phone; classified malware and security attacks with F1 score over 0.91.
Bustamante, A. [51]	2023	Model trained on cloud and deployed on edge via pipeline; classified all anomalies successfully.

## Data Availability

Not applicable.
